# Open-source magnetic system for wireless neuromodulations in vitro and for untethered brain stimulation in vivo

**DOI:** 10.1038/s41598-025-03076-7

**Published:** 2025-05-22

**Authors:** Jun-Xuan Huang, Ping-Hsiang Yen, Chao-Chun Cheng, Yi-Cheng Fang, Po-Han Chiang

**Affiliations:** Institute of Biomedical Engineering, National Yang Ming Chiao Tung University, Hsinchu, Taiwan

**Keywords:** Magnetomechanical stimulation, Magnetic system, Magnetogenetics, Magnetogenetics, Wireless deep brain stimulation, Magnetic neuromodulation, Arduino, Python, Graphic user interface, Open-source., Bionanoelectronics, Neuroscience

## Abstract

In recent years, significant advances have been made in magnetic neuromodulation technologies, enabling the manipulation of deep brain neurons without invasive implants. Wireless approaches, such as those leveraging magnetic nanoparticles and magnetosensitive proteins, have gained considerable attention. Among these, methods requiring low magnetic field density (< 50 mT) and low frequencies (< 20 Hz) show promise for broader applications due to their scalability and energy efficiency. However, the lack of cost-effective, user-friendly instruments for in vitro and in vivo experiments has hindered broader adoption. To address this, we demonstrate an open-source magnetic stimulation system that integrates Arduino-based hardware, electromagnetic coils, and real-time feedback sensors to monitor environmental parameters, including temperature, sound, vibration, and magnetic field density. Additionally, the system employs a closed-loop design, enabling adaptive control of magnetic stimulation based on tracking the subject’s position and environmental feedback. A Python-based graphical user interface (GUI) allows researchers to design and control stimulation protocols while monitoring feedback signals in real-time. The system includes multiple solenoid designs optimized for diverse applications, such as cell culture studies, fluorescence microscopy, and in vivo behavioral experiments, ensuring compatibility across experimental scales. The stability and versatility of the system were evaluated in multiple behavioral paradigms, including light-dark box and place preference tests. This low-cost, easy-access, and flexible platform can facilitate magnetic neuromodulation research and promote accessibility for basic and translational studies in neuroscience and bioelectronics.

**Supplementary Information:**

The online version contains supplementary material available at 10.1038/s41598-025-03076-7.

## Introduction

Magnetic stimulation is the only wireless physical approach capable of activating deep brain regions without the need for implanted hardware or tethered probes. While transcranial magnetic stimulation (TMS) is the most widely used clinical method for magnetic brain stimulation, its effectiveness is limited by the requirement for a strong magnetic field (MF), which restricts stimulation depth to less than 4 cm^1^. Over the last decade, accumulating magnetic neuromodulation approaches have been developed to stimulate neurons using lower MFs, thereby enabling stimulation of deeper brain regions. These approaches can be broadly categorized into two groups (Table 1). The first category includes magnetic nanoparticle-based techniques, which rely on magnetic nanoparticles to convert MFs into heat, electric fields, or mechanical forces, encompassing magnetothermal, magnetoelectric, and magnetomechanical stimulation. The second category consists of magnetosensitive protein-based techniques, in which magnetoreceptive proteins from naturally magnetosensitive species are introduced into the mammalian nervous system to enable magnetic stimulation.

**Table 1:** MF parameters used in previous studies of magnetothermal stimulation, magnetomechanical stimulation, magnetoelectric stimulation, and magnetogenetics. DC MF (mT) refers to the applied static MF, while AC MF (mT) represents the peak value of the AMF. Frequency denotes the operating frequency of the AC MF used in each study.

Technology	DC MF (mT)	AC MF (mT)	Frequency	Reference
Magnetothermal stimulation	-	7–38	160–600 kHz	^2–5,30^
Magnetoelectric stimulation with AMF at kHz range	220	13.6	1 kHz	^8^
Magnetomechanical stimulation	-	20 to 50	0.5 to 20 Hz	^9–12^.
Magnetoelectric stimulation with AMF below kHz range	220	6 to 10	140 to 150 Hz	^6,31^
Magnetogenetics with Electromagnetic-Perceptive Gene (EPG)	41 to 50	-	-	^13–15^
Magnetogenetics with Cryptochrome (CRY)	-	8–10	1–10 Hz	^16^
Magnetogenetics with Cryptochrome (CRY)	100	-	-	^17^

Several magnetic nanoparticle-based approaches have been introduced (Table 1). Magnetothermal stimulation, also known as magnetothermogenetics, is based on the heat generated by magnetic nanoparticles under alternating MFs (AMF) at radio frequencies (160 kHz to 600 kHz)^2–5^. When the thermosensitive cation channel TRPV1 is overexpressed in target neurons, the heat from magnetic nanoparticles can induce neuronal activation wirelessly under radio-frequency AMF. Another approach, magnetoelectrical stimulation, is based on the electric fields generated by nanoparticles when exposed to AMF. Magnetostrictive core-shell nanoparticles are commonly used in this technique, where the magnetostrictive core induces strain on the piezoelectric shell, releasing an electric field that can trigger neuronal activity^6–8^. Unlike magnetothermal approaches, magnetoelectric neuromodulation can be applied without genetic modifications.

Magnetomechanical neuromodulation is a more recent approach developed in the last few years^9–12^. When exposed to a low-frequency AMF (< 20 Hz), magnetic nanomaterials generate mechanical forces that activate mechanosensitive ion channels. Both genetic and non-genetic strategies exist for magnetomechanical stimulation. For instance, overexpression of the mechanosensitive ion channel TRPV4 in target cells allows torque forces from magnetic nanodiscs (~ 250 nm) under weak MF density conditions (< 30 mT, < 20 Hz) to induce Ca²⁺ influx^9^. Similarly, another study demonstrates magnetomechanogenetics by attaching a magnetic particle cluster named m-Torquer to the membrane. When Piezo1, a mechanosensitive ion channel, is overexpressed, mechanical force from m-Torquer under weak MF conditions (< 30 mT, < 10 Hz) can activate neurons^12^. In addition to genetic approaches, magnetomechanical stimulation can also be achieved without gene manipulation. Slightly stronger MF conditions (≤ 50 mT, < 10 Hz) can generate sufficient torque in magnetic nanodiscs to activate intrinsic TRPC channels, which is the mechanosensitive channels naturally expressed in the mammalian brain^10^.

In magnetosensitive protein-based approaches, also called magnetogenetics, involve introducing genes cloned from magnetoreceptive species into the mammalian nervous system to enable magnetic neuronal stimulation (Table 1). Several studies have investigated electromagnetic perceptive genes (EPG) and cryptochrome (Cry), proteins identified in glass catfish, pigeons, and fruit flies. Cells expressing EPG have shown responses to static MFs around 50 mT^13–15^. Cryptochrome (Cry), a circadian clock protein, is implicated in magnetoreception^16^. In Drosophila, Cry is proposed to mediate magnetosensitivity in a blue light-dependent manner, thereby modulating neuronal excitability. When exposed to a 100 mT MF combined with blue light, Cry-expressing neurons exhibit enhanced depolarization and firing activity, supporting its functional role^17^. Although these magnetosensitive properties have been demonstrated, the underlying mechanisms of EPG- and Cry-mediated magnetoreception remain incompletely understood^18,19^.

Additionally, some magnetogenetic constructs, such as magnetoreceptor (MagR) and Magneto, a synthetic fusion of TRPV4 and ferritin, have drawn criticism, as their effects could not be replicated in independent studies^20–24^. Theoretical calculations further suggest that the mechanical force generated by these constructs may be insufficient to elicit neuronal responses^21,25^. Despite these concerns, continued research is necessary to develop reliable magnetosensitive protein-based neuromodulation tools.

Among all magnetic deep brain stimulation (DBS) methods, magnetomechanical, magnetoelectric, and magnetogenetic techniques stand out due to their reliance on low-power, low-frequency MF/AMF (Table 1), enabling scalable magnetic systems for both in vitro and in vivo applications^9–12^. However, an easy-to-use system optimized for magnetic neuromodulation remains lacking. Several studies have introduced magnetic devices for neuromodulation in both in vitro and in vivo contexts^10,12,26^. Still, these systems often lack user-friendliness and adaptability. Some in vivo systems rely on tethered electromagnets mounted to the animal’s head, adding weight and stress that may interfere with behavior^26^. In contrast, an untethered magnetic system using a circular magnet array (CMA) for a large arena employs 6 to 10 bulky 1 T NdFeB magnets (2.5 cm × 3 cm × 4–12.5 cm × 15 cm × 12.5 cm in size)^12^. These require a durable motorized system to rotate the magnets, making the setup cost-prohibitive (Table S1). Although the CMA produces a homogeneous MF of 20 mT at the arena center, this is insufficient in strength and frequency for other techniques like magnetite nanodisc-based magnetomechanical stimulation or magnetogenetics, which require 26 to 50 mT^9,10,26^.

Currently, no commercial system provides a scalable, affordable solution for magnetic neuromodulation. An open-source, low-cost, and accessible system can support wireless DBS in both basic and translational neuroscience, particularly in laboratories with limited budgets or electrical engineering expertise. Several features are critical for widespread adoption: open-source, cost-effective, and easy-to-assemble hardware; integrated feedback sensors; a graphical user interface (GUI) for protocol setup and real-time monitoring; and compatibility with both in vitro and in vivo experiments, including behavioral studies. A closed-loop system capable of dynamically adjusting stimulation based on feedback can further expand application. Here, we introduce a low-cost, untethered magnetic system that satisfies these needs by redesigning magnetic devices reported in earlier work^9,10^. With a maximum MF density of ~ 50 mT, this system supports most magnetogenetic and magnetomechanical stimulation studies across in vitro and in vivo platforms.

## Results

### Magnetic system overview

**Fig. 1:**
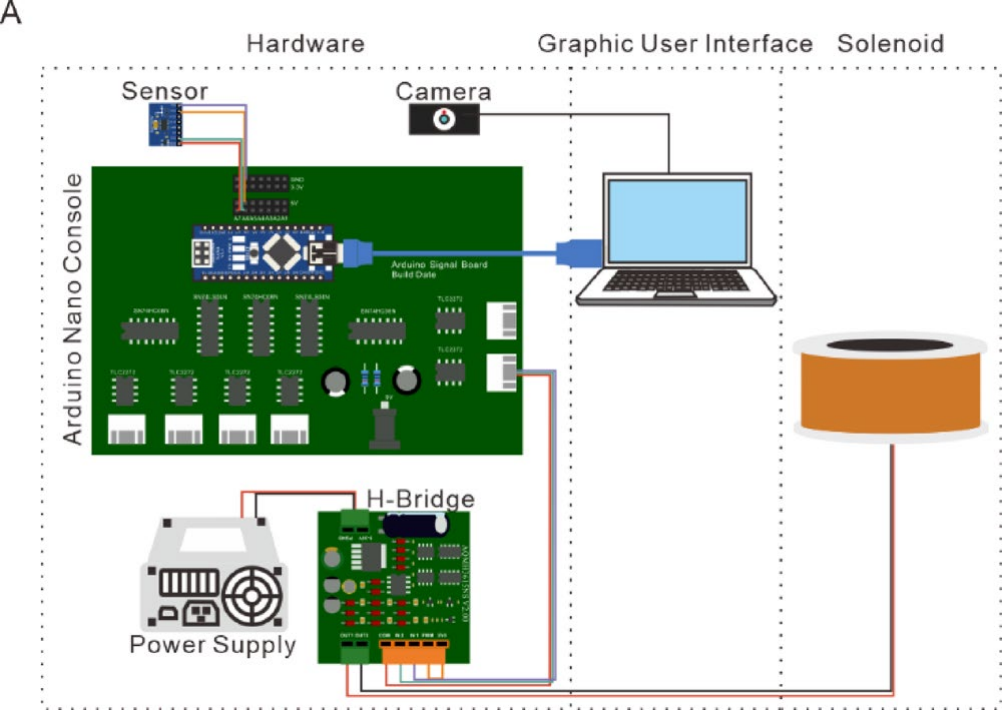
Overview of the magnetic system. (A) The system contains hardware, a GUI, and a solenoid. The hardware includes an Arduino Nano console board, sensors, a camera, an H-bridge, and a power supply. The GUI can send a signal to the Arduino Nano and control the solenoid to generate the AMF.

To improve the accessibility of magnetic stimulation systems, we developed an open-source, user-friendly magnetic platform (Fig. 1). This system consists of three main components: open-source hardware, a Python-based graphical user interface (GUI), and solenoids. The hardware includes an Arduino-based console board, driver boards, power supplies, and four integrated sensors to monitor temperature, sound, vibration, and MF. The GUI, developed in Python, facilitates communication with Arduino, allowing users to configure stimulation protocols, set sensor thresholds, and monitor real-time signal outputs. Instead of using a high-power amplifier, we utilized cost-effective commercial H-bridge circuit boards (AQMH3615 NS, Akelc; Table S2) to control the direction of the MF within each solenoid (Fig. 1). The H-bridge drivers manipulate the AMF direction based on input signals from the console board. This modular design allows for integrating various solenoid configurations, making the system adaptable to different neuromodulation applications.

### Arduino-based hardware and firmware

**Fig. 2:**
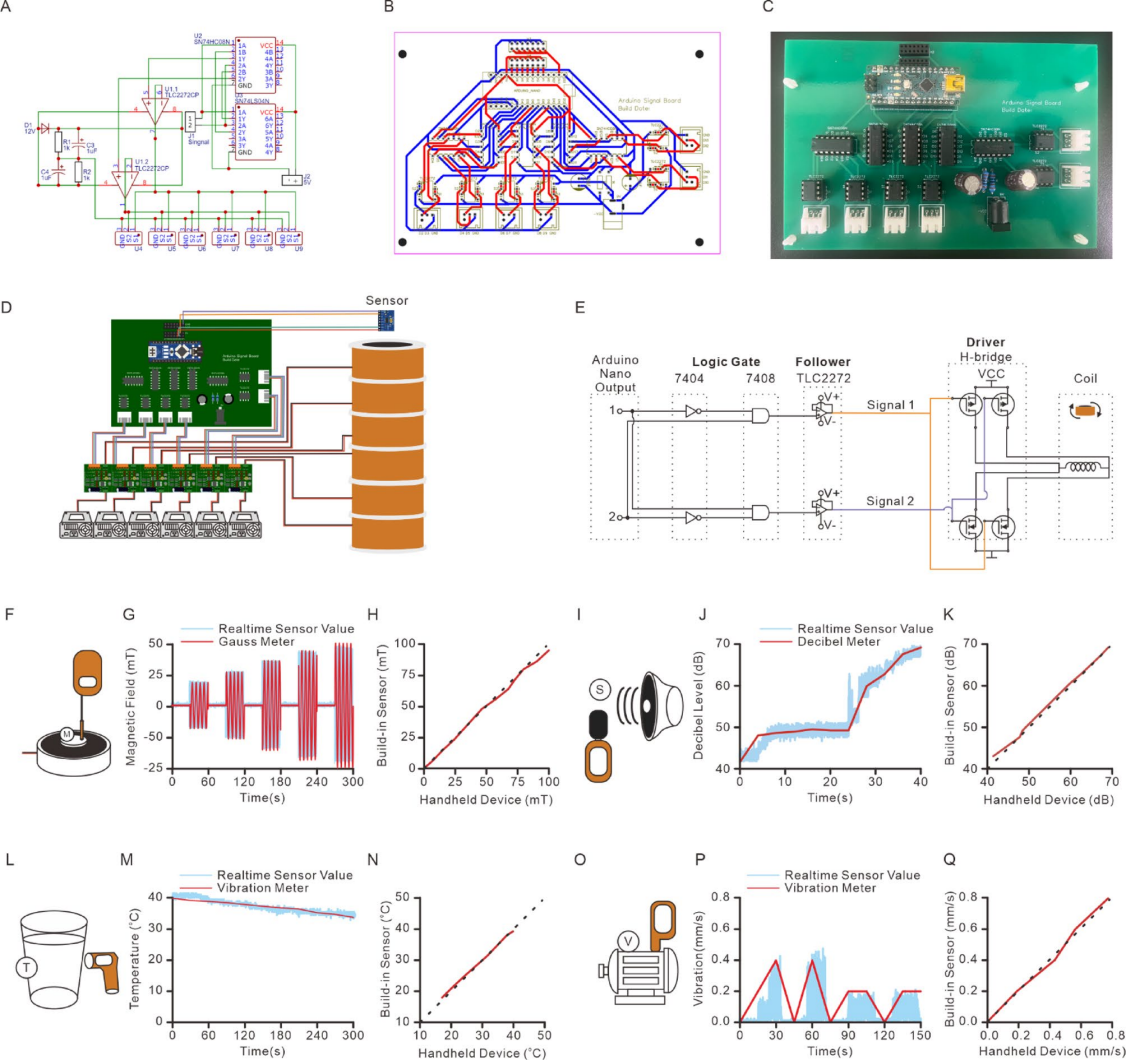
The hardware design for magnetic system. (**A**) The circuit diagram of the console board. (**B**) The layout of the console board. (**C**)The photo of the console board. (**D**) The schematic of console board demonstrates the setup to control six coils and monitors the condition of the coils. (**E**) The logic circuit diagram of the console board demonstrates the signal from Arduino Nano to diver and the direction of the MF changed. (**F**) The schematic of MF measurement on an electromagnet with the Hall sensor and the handheld Gauss meter. (**G**) The MF intensity change measured by the Hall sensor and the handheld Gauss meter during the AMF application by an electromagnet. (**H**) The correlation between the field intensity measured by Hall sensor and the handheld Gauss meter. (**I**) The schematic of the decibel measurement near the speaker with the decibel sensor and the handheld decibel meter. (**J**) The decibel change measured by the decibel sensor and the handheld decibel meter during the volume change of the speaker. (**K**) The correlation between the recording from the decibel sensor and the handheld decibel meter. (**L**) The schematic of the temperature measurement on the beaker with hot water by the temperature sensor and the infrared thermometer. (M) The temperature change measured by the temperature sensor and the infrared thermometer during the temperature change. (**N**) The correlation between the temperature measured by temperature sensor and the infrared thermometer. (**O**) The schematic of the acceleration measurement on the motor with the acceleration sensor and the handheld vibrometer. (**P**) The acceleration change measured by the acceleration sensor and the handheld vibrometer during the on and off of a motor. (**Q**) The correlation between the acceleration measured by the acceleration sensor and the handheld vibrometer.

This open-source magnetic system features an Arduino-based console board that delivers control signals and receives sensor feedback (Fig. 2). The system utilizes an Arduino Nano, a compact and cost-effective version of the widely adopted Arduino Uno, making it an ideal choice for integration. The console board outputs 5 V signals through 12 digital pins, which are organized into six pairs (Fig. 2A-C). Each pair controls the direction of the MF in one solenoid via an H-bridge driver (Fig. 2D). This configuration enables the system to control up to six solenoids simultaneously, expanding its applicability to larger experimental arenas. The board also includes eight analog input pins for sensor data acquisition and six additional digital pins, which can send signals to external devices, such as a digitizer for synchronizing stimulation timing (Fig. 2A-C). To prevent short circuits in the H-bridge drivers, both hardware and firmware protections are implemented. Firmware protection ensures that paired output signals are delivered at staggered time intervals, avoiding conflicting high-level outputs (Fig. 2E). Hardware protection is provided through logic gates on the console board, which prevent signal conflicts that could damage the driver circuitry (Fig. 2E). The SN74HC08 N IC, containing four independent AND gates, and the SN74HC04 N IC, containing six independent NOT gates, are used to enhance signal stability and reliability (Table S3).

The system integrates four types of sensors to monitor solenoid performance during magnetic stimulation: MF density, sound, vibration, and temperature (Fig. 2F-Q, Table S3). These sensors connect to the Arduino Nano via analog inputs, providing real-time feedback on environmental conditions. The SS495 A magnetic sensor measures MF density and direction, ensuring that the applied field reaches the preset levels during stimulation. It operates at low power (7 mA, 5 V) and has a compact form factor (Fig. 2F; Table 2). The MF values recorded by the SS495 A sensor strongly correlate (*r* = 0.994) with those from a handheld Gaussmeter (FM302, Projekt Elektronik) (Fig. 2G-H). The Bland-Altman analysis indicates an upper limit of agreement (LoA) of 3.33, a lower LoA of −2.86, and a bias of 0.24 (Fig. S1A).

**Table 2:** The properties of sensors in this study.

Sensor	Part number	Supply Voltage (V)	Supply Current (mA)	Detection Range
Magnetic	SS495 A	4.5–10.5	7	−67mT − 67 mT
Temperature	TMP36GT9Z	2.7–5.0	0.05	−40–125 °C
Sound	SEN0232	3.3–5.0	14	30–130 dB
Vibration	ADXL345	2.0–3.6	0.14	± 16 g

Monitoring unwanted conditions such as temperature increases, noise, and solenoid vibrations is crucial to prevent additional stress in animals and to ensure experimental integrity. A sound sensor (SEN0232) was integrated into the system to detect solenoid-generated noise (Fig. 2I; Table 2, S3). This sensor operates with low power consumption and requires minimal external components. The SEN0232 has a sensitivity range of 31.5 Hz to 8.5 kHz, making it suitable for detecting high-frequency harmonic components of coil-generated noise. Although it may not optimally detect low-frequency noise below 31.5 Hz, this limitation is not significant in rodent studies, as rats and mice have poor auditory sensitivity in this range. Studies indicate that rats and mice primarily detect sounds starting at 500 Hz and 1 kHz^27^, making ultra-low-frequency noise detection unnecessary for behavioral experiments. The noise measurements obtained with SEN0232 strongly correlate with those from a decibel meter (UT353, Uni-T) (*r* = 0.999) (Fig. 2J-K). The Bland-Altman analysis shows an upper LoA of 0.77, a lower LoA of −0.98, and a bias of −0.11 (Fig. S1B).

A TMP36GT9Z temperature sensor was used to monitor temperature changes during MF applications (Fig. 2L; Table 2, S3). This sensor features low self-heating and low power consumption, making it suitable for long-term monitoring. The temperature recorded by TMP36GT9Z correlates well with values from a handheld infrared thermometer (IR-829, Phils Trust Medical Center) (*r* = 0.998) (Fig. 2M-N). The Bland-Altman analysis shows an upper LoA of 0.94, a lower LoA of −0.71, and a bias of 0.11 (Fig. S1C).

A 3-axis accelerometer (ADXL345) was used to measure vibration levels, providing high-resolution (13-bit) acceleration data with low power consumption (Fig. 2O; Table 2, S3). The vibration data from ADXL345 closely matches the measurements from a vibration detector (GM63 A, Benetech) (*r* = 0.997) (Fig. 2P-Q). The Bland-Altman analysis indicates an upper LoA of 0.03, a lower LoA of −0.02, and a bias of 0.01 (Fig. S1D). These results confirm that the sensor inputs in this system are highly consistent with standard handheld devices, ensuring accurate monitoring of solenoid activity.

During experiments, the system monitors solenoid status in real time and interrupts stimulation if temperature, noise, or vibration exceed preset limits. Sensor detection runs at 20 Hz continuously, with additional sensor readings triggered when MF state changes occur. During AMF applications, the system performs two extra sensor readings per stimulation cycle to ensure stability. The Arduino firmware receives stimulation parameters, sensor thresholds, and real-time commands from the Python-based software, and returns sensor values and timing feedback. The Arduino’s internal clock oscillator regulates stimulation timing. The result reveals that when all sensor detection features are activated, signal timing remains stable at frequencies below 200 Hz (Fig. S2 A). At higher frequencies, the sensor detection duration slightly impacts signal output timing; however, when sensor detection are disabled, stimulation remains stable above 200 Hz (Fig. S2B). Since most magnetomechanical, magnetoelectric, and magnetosensitive protein-based techniques operate below 150 Hz (Table 1), this system reliably supports neuromodulation applications.

### Open-source software design

**Fig. 3:**
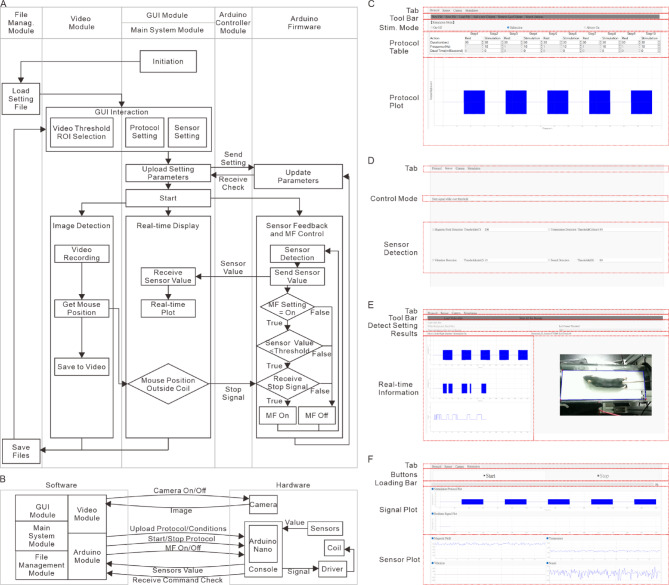
Overview of the GUI. (**A**) The flowchart of modules in the GUI system and firmware in Arduino. demonstrates the communications between ” File Management Module”, “Main System Module”, “Arduino-Controller Module”, “Video Module”, and Arduino firmware (**B**) A schematic drawing demonstrates the communications between the software, firmware and hardware a. (**C**)The “Protocol” page of the GUI, which was used for setting up the stimulation condition and protocols (**D**) The “Sensor” page of the GUI, which was used for setting up the sensors and the limitation. (**E**) The “Camera” page of the GUI, which was used for setting up the ROI, threshold, and other parameters for close-loop stimulation. This page was also used for real-time monitoring of MF and behavior. (**F**) The” Stimulation” page of the GUI, which was used for controlling the stimulation and monitoring the feedback from the sensors during the stimulation.

The software interface was developed by using Python 3.8.8 and PyQt5 5.15.7 to provide a fully functional GUI for controlling the magnetic system. Communication between the Arduino and the computer is established using pyserial 3.5, enabling real-time data exchange between hardware and software components. The software architecture consists of five primary modules: the GUI module, main system module, video module, Arduino-controlling module, and file management module (Fig. 3A). The system enables closed-loop control of MF applications by integrating real-time sensor feedback and behavioral tracking, allowing magnetic stimulation to be dynamically adjusted based on environmental conditions and subject movement.

The GUI module serves as the primary interface, providing researchers with an intuitive platform for configuring stimulation protocols, setting sensor thresholds, and monitoring experimental conditions in real time. The main system module executes stimulation protocols, manages sensor input thresholds, and processes real-time video tracking data. It communicates with the Arduino-controlling and video modules to ensure synchronized operation. This module performs three main tasks: setting up experimental parameters such as stimulation protocols, sensor limitations, and video detection thresholds; controlling Arduino and camera functions; and receiving sensor and video feedback for closed-loop control of MF applications. The Arduino-controlling module facilitates communication between the computer and Arduino Nano. It transmits stimulation parameters and sensor thresholds while continuously receiving feedback from the MF, sound, vibration, and temperature sensors (Fig. 3B). This module ensures stimulation is applied only when environmental conditions remain within predefined limits. It also manages sensor start/stop functions, receives sensor data from the Arduino, and controls stimulation initiation and termination. The video module detects subject movement in real time using video tracking algorithms (Fig. 3A). Users can set detection parameters, such as pixel intensity thresholds and regions of interest (ROIs). This module processes video input, identifies the position of subject, and transmits data to the main system module to determine stimulation conditions. It also records video data for post-experiment analysis. The file management module ensures that stimulation protocols, sensor data, and behavioral tracking results are stored and retrievable for future use. Data are saved in CSV format, allowing easy integration with external analysis tools. This feature enables researchers to reproduce experiments accurately and efficiently analyze sensor feedback.

To achieve closed-loop magnetic neuromodulation, the main system module determines when AMFs are applied based on three conditions (Fig. 3A). First, the system ensures that the stimulation protocol is set to “ON” during the programmed time intervals. Second, all sensor feedback values (MF, sound, vibration, and temperature) must remain within preset thresholds. Third, real-time video tracking algorithm confirms that the subject is within the target stimulation area. If all conditions are met, AMF stimulation begins; otherwise, the MF remains inactive. By integrating sensor feedback, real-time tracking, and programmable protocols, this system provides adaptive neuromodulation with precise control of MF delivery during behavioral experiments. The software design ensures flexibility and ease of use, allowing researchers to customize stimulation parameters for a wide range of in vitro and in vivo studies.

### Design of GUI

A graphical user interface (GUI) was developed to facilitate magnetogenetics and magnetic DBS experiments without requiring programming expertise. The GUI, built using PyQt5, provides an intuitive and user-friendly platform for configuring stimulation parameters, setting sensor thresholds, monitoring real-time data, and enabling closed-loop control of MF applications. It consists of four main tabs, each designed for a specific function: stimulation protocol settings, sensor settings, video recording and feedback, and real-time monitoring (Fig. 3C-F).

The first tab is dedicated to stimulation protocol settings (Fig. 3C; Table S4). The top block contains a toolbar with essential functions such as uploading, saving, and loading stimulation protocols. While the system can automatically detect and connect to the Arduino, a “Search Arduino” button allows for manual connection. Below, the stimulation protocol section enables users to configure **either** unidirectional **or** bidirectional MF stimulation. The protocol table provides options for defining stimulation and resting periods, MF frequencies, and dead times between signals, with the ability to add or remove multiple periods (Fig. 3C; Table S4). A protocol plot at the bottom visually represents the programmed stimulation sequence, helping users verify the setup before execution.

The second tab is designed for sensor configuration and threshold settings (Fig. 3D). The control mode section allows users to specify whether MF stimulation should stop when sensor values exceed predefined thresholds. The sensor settings block enables users to select active sensors and define their limits for MF intensity, sound, vibration, and temperature. If any sensor detects unexpected fluctuations, the system automatically pauses stimulation to prevent unintended experimental influences.

The third tab (Fig. 3E) handles video tracking and real-time feedback, using OpenCV 4.5.5.64 for image processing. The detection settings section allows users to define the region of interest (ROI) and adjust the pixel intensity threshold to determine the mouse position. The interface includes buttons for starting ROI selection, loading pre-recorded videos, resetting detection areas, and emergency stopping. The detection mode settings provide options for defining the behavior test type, target color (black or white), and stop conditions for the MF application. The real-time video feedback panel continuously updates the mouse’s chamber position and stimulation status, while the lower panel displays plots of the protocol, real-time signal outputs, and the mouse’s movement data.

The fourth tab (Fig. 3F) provides real-time monitoring and manual stimulation control. The start/stop control block allows users to activate or deactivate MF stimulation. Below this, a stimulation protocol plot visualizes both the preset protocol and real-time signal output, ensuring that the system operates as expected. The four data plots at the bottom display MF intensity, vibration levels, sound levels, and temperature in real-time, allowing researchers to monitor environmental conditions throughout the experiment.

This GUI ensures straightforward control over experimental settings, real-time monitoring of system performance, and seamless integration of behavioral tracking with magnetic stimulation, making it accessible for researchers without programming expertise. By providing a streamlined interface for experimental setup, execution, and monitoring, the GUI enhances the usability and reproducibility of magnetic neuromodulation studies. The system’s usability was further evaluated through a standard usability questionnaire^28^, which yielded a score of 86.9 ± 4.6 (*n* = 9, Table S5). It shows that the interface is highly intuitive, accessible, and easy to use for researchers conducting magnetic neuromodulation studies.

### Design of solenoids for various applications

**Fig. 4:**
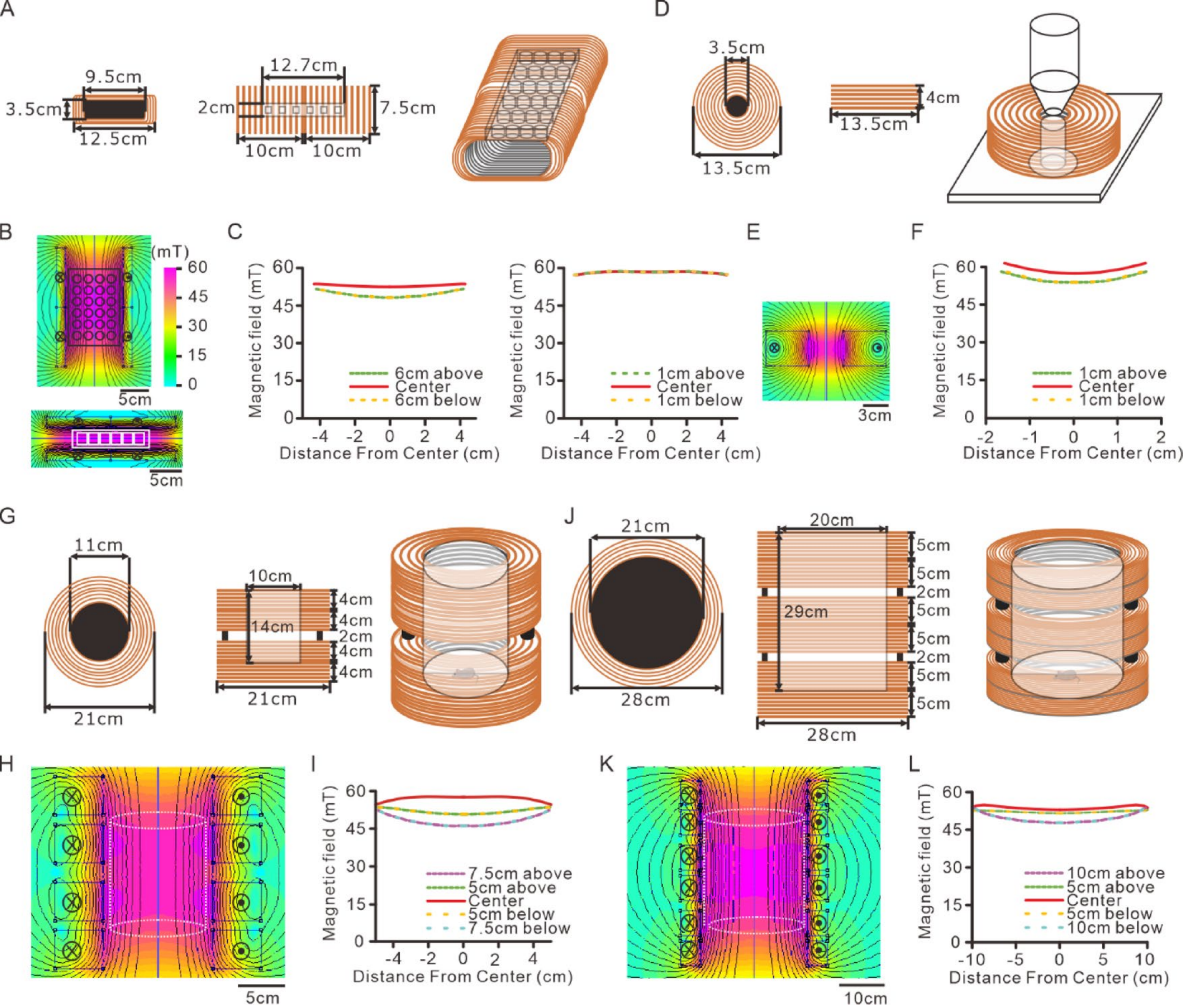
The FEMM simulation of coils for different purposes. (**A**) Multi-view drawing of the coils for multi-well culture dish. Left, the front view. Middle, the side view. Right, the oblique view. (**B**) The heat map of FEMM simulation of the magnetic strength in the same coils as (**A**). Top, the simulation with top view of the coils. Bottom, the simulation with side view of the coils. (**C**) Left, the simulated MF intensity at center line, and 5 cm from the center of the top view simulation in (**B**). Right, the simulated MF intensity at center line, and 1 cm from the center of the side view simulation in (**B**). (**D**) Multi-view drawing of the coil with 3.5 cm inner diameter. Left, the front view. Middle, the side view. Right, the oblique view. (**E**) The heat map of the magnetic strength by FEMM simulation of sagittal plane of the same coil as (**D**). (**F**) The simulated MF intensity at center line, and 1 cm from the center in (**E**). (**G**) Multi-view drawing of the coils with 10 cm arena. Left, the front view. Middle, the side view. Right, the oblique view. (**H**) The heat map of the magnetic strength by FEMM simulation of sagittal plane of the same coils as (**G**). (**I**) The simulated MF intensity at center line, at 5 cm from the center, and at 7.5 cm from the center in (**H**). (**J**) Multi-view drawing of the coils with 20 cm arena. Left, the front view. Middle, the side view. Right, the oblique view. (**K**) The heat map of the magnetic strength by FEMM simulation of sagittal plane of the same coils as (**J**). (**L**) The simulated MF intensity at center line, at 5 cm from the center, and at 10 cm from the center in (**K**).

This magnetic system was designed to accommodate various applications in magnetic neuromodulation by supporting a range of solenoid configurations. The commercial H-bridge circuit boards (AQMH3615 NS, Akelc; Table S2) used in this system have a current limit of 20 A, a voltage range from 9 to 36 V, and a maximum power output of 380 W. When combined with a 960 W power supply capable of delivering a maximum current of 20 A and voltage of 48 V (HJS-1000-0-48 V, Yueqing Zhijiu; Table S2), the system provides sufficient power for various magnetic apparatus designs. The alternating or static MF density within 50 mT is applicable for magnetomechanical stimulation, magnetoelectric stimulation, and magnetogenetics (Table 1). Here, we present four different solenoid configurations for in vitro and in vivo applications, each designed to operate at AMF densities below 50 mT (Fig. 4).

For in vitro applications, an air-core rectangular cuboid coil was designed to stimulate cultured cells in multiple-well culture dishes. This coil has an inner height of 3.5 cm, an inner width of 9.5 cm, an outer height of 7.5 cm, an outer width of 12.5 cm, and a length of 20 cm (Fig. 4A). The multiple-well culture dish, measuring 8.5 cm in width and 12.7 cm in length, is positioned at the center of the coil (Fig. 4B). The 24 culture wells, spanning a 5.5 cm width and 9.7 cm length, are within the homogeneous MF region of the coil. Using Finite Element Method Magnetics (FEMM) software, we simulated the MF distribution in both horizontal and sagittal planes. At 3.2 A, the MF density in the horizontal plane exhibits 5.18% homogeneity, with values ranging from 55.7 mT to 58.7 mT (Fig. 4B-C). In the sagittal plane, the homogeneity is 13.62%, with MF density values ranging from 45.8 mT to 52.7 mT (Fig. 4B-C). This coil configuration enables direct stimulation of neurons in multiple-well culture dishes without requiring sample transfers, making it suitable for long-term studies such as nerve regeneration or neuronal growth research.

For fluorescence microscopy applications, a solenoid coil was designed with an inner diameter of 3.5 cm, an outer diameter of 13.5 cm, and a height of 4 cm (Fig. 4D). Simulation with a 2.2 A current in the sagittal plane shows that the MF density within a 2 cm height and 2 cm width at the center of the coil had 3.77% homogeneity, with field densities ranging from 58.0 mT to 60.2 mT (Fig. 4E-F). The MF density homogeneity over a 2 cm width is 8.52%, with field values ranging from 55.24 mT to 60.21 mT. A custom-designed sample holder is incorporated into the coil to ensure consistent and reproducible stimulation during imaging experiments.

In addition to in vitro applications, the system includes large solenoid-based magnetic apparatuses for in vivo studies. A solenoid array was designed to accommodate a 10 cm diameter behavioral arena consisting of four solenoids with an inner diameter of 11 cm, an outer diameter of 21 cm, and a height of 4 cm each (Fig. 4G). The solenoids were arranged with gaps of 1 cm, 2 cm, and 1 cm between them. Simulation using a 6 A current shows that within a 5 cm height and 10 cm diameter region at the center, MF density homogeneity is 10.08%, with field densities ranging from 52.3 mT to 57.8 mT (Fig. 4H-I). Over a 10 cm height, homogeneity is 11.5%, with MF density values ranging from 52.0 mT to 58.2 mT. At 15 cm height, homogeneity decreases to 22.9%, with field densities ranging from 46.0 mT to 58.2 mT.

For larger in vivo studies, an expanded solenoid system was designed for a 20 cm diameter arena, incorporating six solenoids, each with a height of 5 cm, an inner diameter of 21 cm, and an outer diameter of 28 cm (Fig. 4J). The solenoids were stacked with gaps of 1, 2, 1, 2, and 1 cm between them. Simulation with a 7 A current shows that within a 5 cm height and 20 cm diameter region at the center, MF density homogeneity is 5.61%, with densities ranging from 52.5 mT to 55.5 mT. Within a 10 cm height, homogeneity is 7.43%, with MF density values from 51.6 mT to 55.5 mT. Within a 15 cm height, homogeneity is 10.31%, with densities from 50.1 mT to 55.5 mT. Finally, at 20 cm height, homogeneity was 14.52%, with field values from 47.98 mT to 55.52 mT.

These solenoid configurations provide adaptable solutions for a variety of experimental conditions, ensuring controlled MF density exposure for both in vitro and in vivo magnetic neuromodulation studies. The ability to modify solenoid designs allows researchers to tailor the system for specific study requirements, thereby enhancing its versatility and applicability in neuroscience research.

### Measurement of magnetic apparatus

**Fig. 5:**
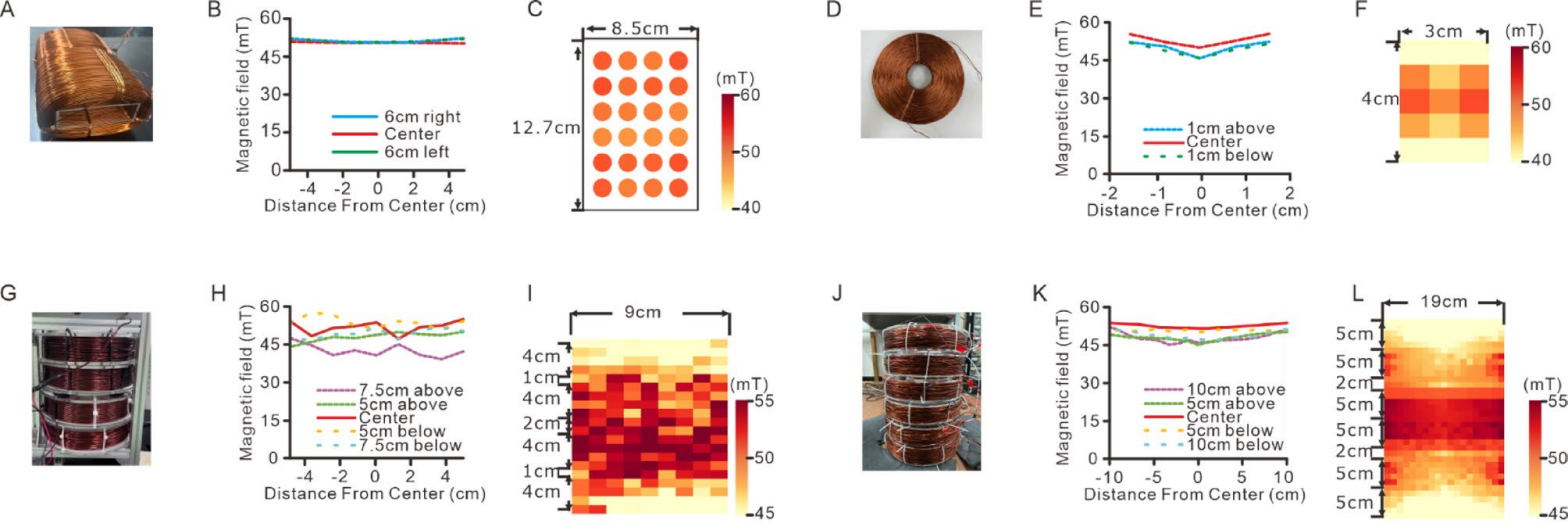
The properties of fabricated coils for different purposes. (**A**) Photo of the coils for a multi-well culture dish. (**B**) The MF intensity at the middle line and at 6 cm from the center in the same coils as (**A**). (**C**) The heat map of MF strength was measured at the location of each well of the 24-well dish in the coils. (**D**) Photo of the coil with 3.5 cm inner diameter. (**E**) The MF intensity at the middle line and at 1 cm from the center in the same coil as (**D**). (**F**) The heat map of MF strength measured in the same coil as (**D**). (**G**) Photo of the coils for 10 cm diameter arena. (**H**) The MF intensity at the middle line, at 5 cm from the center and at 7.5 cm from the center in the same coils as (**G**). (**I**) The heat map of MF strength measured in the same coils as (**G**). (**J**) Photo of the coils for 20 cm diameter arena. (**K**) The MF intensity at the middle line, at 5 cm from the center, and at 10 cm from the center in the same coils as (**J**). (**I**) The heat map of MF strength measured in the same coils as (**J**).

To evaluate the performance of the designed magnetic apparatuses, we constructed and characterized the solenoids to assess their MF density distribution and homogeneity (Fig. 5). The experimental measurements are compared with theoretical values calculated using the Biot*-*Savart law to validate the accuracy and consistency of the system (see supplementary information). For the multiple-well culture dish configuration, each solenoid has a resistance of 6.9 Ω and an inductance of 50 mH (Fig. 5A). When applying ~ 32 V and ~ 3.6 A to each coil, the MF densities at the locations of the individual wells within the 24-well plate are also highly uniform, with a homogeneity of 6.06%, ranging from 49.4 mT to 52.5 mT (Fig. 5B-C). These results indicate the apparatus is well-suited for simultaneously stimulating neuronal cultures in vitro. The measured MF density values are slightly lower than the theoretical prediction of 64.8 mT, likely due to real-world coil imperfections (Table S6).

For fluorescence microscopy applications, a 3.5 cm air-core coil was constructed with a resistance of 7 Ω and an inductance of 50 mH (Fig. 5D). With applying 35 V and 2.7 A, the MF density at the center of the coil remains highly homogeneous, with 5.03% variation within a 0.8 cm height and 3 cm diameter, ranging from 50.1 mT to 52.7 mT (Fig. 5D-F). Although the MF density decreases more rapidly along the vertical axis, the system maintains 15.63% homogeneity over a 2.4 cm height at the center, with values ranging from 45 mT to 52.7 mT. The use of a custom sample holder ensures precise positioning of specimens for consistent magnetic stimulation. The measured MF densities are in close agreement with theoretical calculations, which predicted a central MF density of 55.3 mT (Table S6).

For in vivo applications, four solenoids were constructed for the 10 cm diameter arena (Fig. 4G). Each solenoid has a resistance of approximately 1 Ω and an inductance of ~ 9 mH. When applying ~ 23 V and ~ 7.2 A to each coil, the MF density exhibits 20.80% homogeneity within a 5 cm height and 10 cm diameter at the center, ranging from 47.4 mT to 58.4 mT. Over a 10 cm height, homogeneity remains at 21.19%, with MF density values between 47.2 mT and 58.4 mT (Fig. 5G-I). At a height of 15 cm, the homogeneity decrease to 29.13%, with MF densities between 43.3 mT and 58.4 mT. At 20 cm height, the homogeneity drops to 57.05%, with MF densities ranging from 30.2 mT to 58.4 mT. These experimental values are comparable to theoretical predictions, which estimated an MF density of 56.2 mT at the center (Table S6).

For the larger 20 cm diameter arena, six solenoids were constructed based on the design in Fig. 4J (Fig. 5J). Each solenoid has a resistance of ~ 1.6 Ω and an inductance of ~ 13 mH. When applying ~ 40 V and ~ 9.5 A to each coil, the MF density exhibits 7.80% homogeneity within a 5 cm height and 20 cm diameter at the center, ranging from 50.5 mT to 54.6 mT. Over a 10 cm height, homogeneity is 19.05%, with MF densities between 45.5 mT and 55.4 mT (Fig. 5K-L). At 15 cm height, homogeneity is 19.36%, with MF densities from 45.5 mT to 55.4 mT. Finally, at 20 cm height, homogeneity is 23.41%, with MF densities ranging from 43.6 mT to 55.4 mT. These measured values are lower than the theoretical prediction of 68.2 mT, likely due to manufacturing variability and material imperfections (Table S6).

A mouse is ~ 8 cm in length from nose to tail base, ~ 3 cm in width, and ~ 3 cm in height when crawling. The size and MF density distribution of the apparatuses for both the 10 cm and 20 cm arenas are sufficient to support most in vivo applications of wireless neuromodulation. The range of homogeneous MF density is extensive enough to ensure effective stimulation of freely moving animals, including applications involving magnetic nanoparticle-based stimulation and magnetogenetics. These results confirm the system provides a stable and adaptable platform for in vivo neuromodulation studies.

### The stability of the magnetic system for in vivo studies

**Fig. 6:**
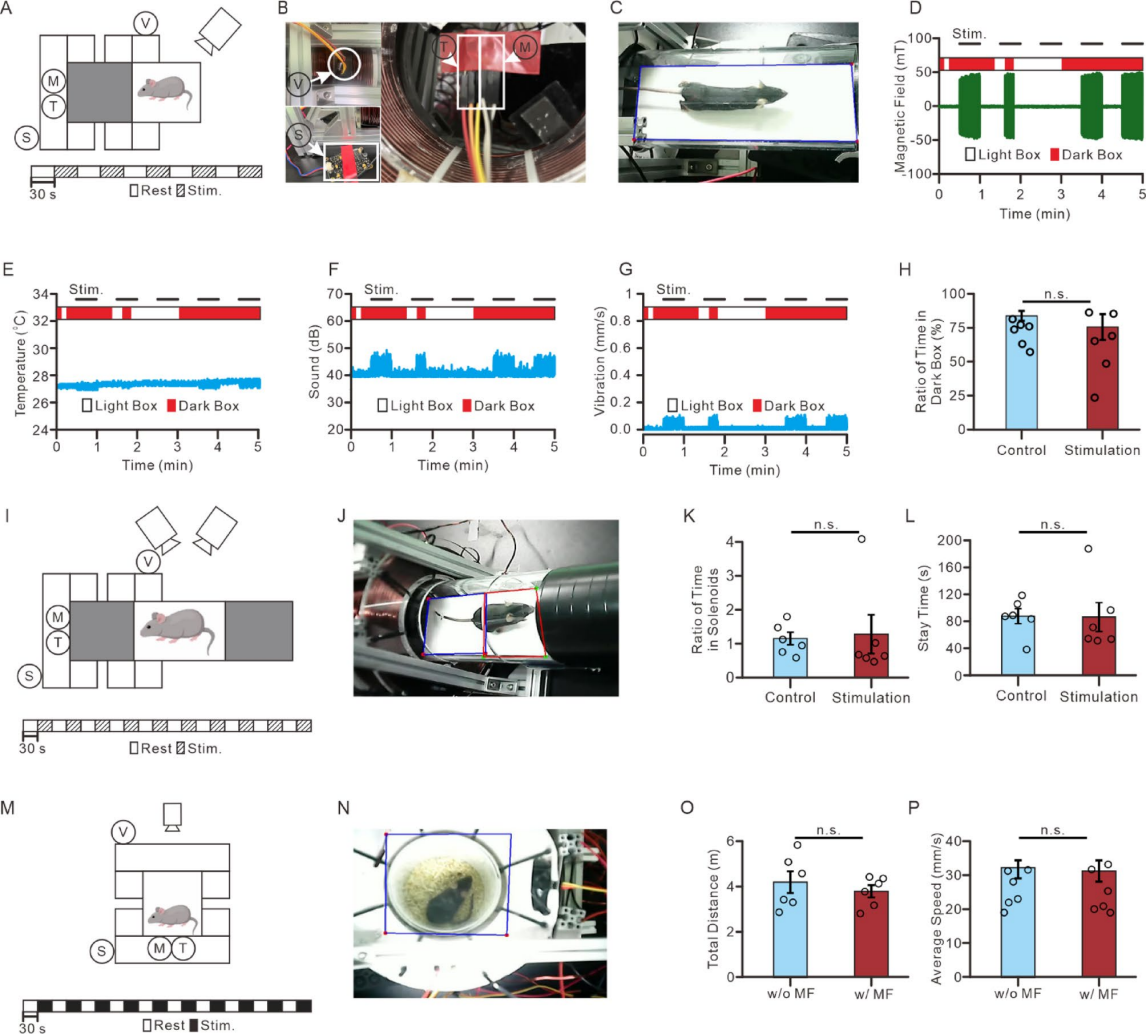
The performance of magnetic apparatus with 10 cm diameter arena in mice behavioral test. (**A**) The schematic and the timeline of the light-dark box behavioral test demonstrates the setting in the test. (**B**) The photos of the position of the sensors. (**C**) The photo of the light-dark box during the behavioral test. (**D**) A representative trace of the MF changes in the light-dark box behavioral test demonstrates that the MF corresponded to the mouse position. (**E**) A representative trace of the temperature changes in the light-dark box behavioral test. (**F**) A representative trace of the decibel changes during the light-dark box behavioral test. (**G**) A representative trace of the vibration changes during the light-dark box behavioral test. (**H**) The ratio between the time of mice spent in the dark box and the total duration of test (*n* = 6). (**I**) The schematic and the timeline of the place preference behavioral test. (**J**) The photo of mouse during the place preference behavioral test. (**K**) The ratio between the time of mice spent in the chamber with the coils and the time of mice spent in the chamber without the coils during the place preference test (*n* = 6). (**L**) The total time duration of mice spent in the chamber with coils the place preference test (*n* = 6). (M) The schematic and the timeline of the free-moving behavioral test. (**N**) The photo of mice during the free-moving test. (**O**) The total distance mice traveled during the test with and without MF application (*n* = 6). (**P**) The average speed of the mice during the test with and without MF application (*n* = 6). n.s., no significance; Wilcoxon signed-rank test.

To evaluate the stability and applicability of the open-source magnetic system for in vivo experiments, we assessed its effects on wild-type mice in three different behavioral paradigms. These experiments determined whether switching on the magnetic apparatus influenced behavior due to sound, vibration, temperature changes, or direct exposure to MF. The first experiment involved a light-dark box test, a widely used behavioral assay to measure anxiety-like responses in mice. Wild-type mice typically prefer the dark compartment and avoid the brightly lit area. To determine whether exposure to the magnetic apparatus caused aversive effects, we positioned the dark box within the 10 cm solenoid system and placed multiple environmental sensors around the setup (Fig. 6A-C). A three-axis accelerometer was mounted on top of the solenoid array to measure vibration. At the same time, a hall sensor, thermometer, and decibel sensor were placed along the dark box wall to monitor MF density, temperature changes, and noise levels, respectively (Fig. 6B).

Magnetic stimulation was applied at 50 mT, 10 Hz, with 30-second stimulation periods alternating with 30-second rest periods for five cycles (Fig. 6A). The system was programmed to activate MF density only when mice entered the dark box, ensuring that exposure was behavior-dependent. Real-time tracking confirms that MF density was not applied if the mice remained in the light box (Fig. 6D). Additionally, temperature, vibration, and noise fluctuations remain minimal throughout the stimulation period (Fig. 6E-G). Behavioral analysis reveals no significant difference in the proportion of time spent in the dark box between control mice (71.5 ± 4%) and stimulated mice (63.1 ± 10%) (Fig. 6H). These results indicate that activating the magnetic apparatus did not produce aversive effects due to environmental factors such as noise, vibration, or temperature changes.

To investigate whether MF density influenced place preference behavior, we conducted a real-time three-chamber place preference test, a paradigm used to assess reward and aversion learning. The magnetic apparatus was placed in one of the three chambers, and magnetic stimulation (50 mT, 10 Hz) was applied in 30-second intervals alternating with 30-second rest periods for ten cycles (Fig. 6I-J). Similar to the light*-*dark box test, closed-loop magnetic stimulation was delivered only when the mouse entered the stimulation chamber. A secondary camera was installed to detect entry into the adjacent chamber (Fig. 6I). There are no significant difference in the relative time spent in the stimulation chamber compared to the control chamber between stimulated mice (1.2 ± 0.6) and control mice (1.1 ± 0.2) (Fig. 6K). Total time spent in the stimulation chamber is also similar between control mice (87.0 ± 11.2 s) and stimulated mice (85.7 ± 21.5 s) (Fig. 6L). These findings confirm that magnetic stimulation did not affect place preference behavior.

Finally, we conducted a freely moving locomotion test in a vertical magnetic apparatus to determine whether AMF exposure altered general locomotor activity (Fig. 6M-N). Behavioral parameters were analyzed before and during MF density application, allowing for a within-subject comparison. There are no significant differences in total distance traveled between baseline conditions (4.2 ± 0.5 m) and stimulation periods (3.8 ± 0.3 m) (Fig. 6O). Likewise, average movement speed remains unchanged between non-stimulation periods (32.3 ± 2.6 mm/s) and stimulation periods (31.3 ± 3.1 mm/s) (Fig. 6P). These data indicate that activating the magnetic apparatus did not disrupt locomotor activity, supporting its suitability for in vivo behavioral studies. Collectively, these findings show that the magnetic apparatus does not induce stress, aversion, or behavioral disruption due to environmental factors such as noise, vibration, or temperature. Which makes it highly adaptable for various behavioral neuroscience applications, ensuring precise and controlled neuromodulation without interfering with natural behaviors.

## Discussion

Here, we present an open-source, user-friendly, and cost-effective magnetic system. The MF density reaches up to 60 mT, and the frequency extends up to 20 Hz, both of which fall within the range required by the latest magnetic neuromodulation technologies. We demonstrate the magnetic apparatuses suitable for both in vitro and in vivo experiments, which can be used for neuromodulation in culture dishes, fluorescence microscopes, and behavioral studies in large arenas. In our platform, the magnetic density is adjusted by limiting the current or voltage of the power supply to the driver. Calibration of the MF should be performed before application. Moreover, researchers can monitor real-time magnetic intensity using the built-in Hall sensor. Although this platform cannot dynamically vary MF density during operation without a power amplifier, this functionality is unnecessary for most experiments. Beyond the demonstrated solenoid designs, the system can be adapted to support coils with higher MF density and extended frequency ranges, allowing broader neuromodulation applications. Feedback sensors ensure that stimulation remains within defined limits.

The circuit design of the console board plays a crucial role in the system’s stability and performance. The use of 7404 and 7408 logic gates prevents signal conflicts that could cause unintended short circuits in the H-bridge drivers. Integrating a TLC2272 voltage follower effectively isolates power to the H-bridge, reducing noise and ensuring stable signal transmission. The current design supports H-bridge drivers operating with 5 V input signals, which reliably drive solenoids up to 20 cm in diameter in an arena. If future applications require higher voltage outputs to drive different H-bridge configurations with increased power demands, modifications incorporating open-collector buffers, such as the 7407 IC, may be necessary to enhance voltage flexibility and improve isolation between control and power circuits. Additionally, an alternative DRV8701-based driver design could be explored to allow integration with high-power external MOSFETs in an H-bridge configuration, enabling higher-current solenoid operation. Nevertheless, the current design offers a stable, efficient, and cost-effective solution that addresses limitations of previous neuromodulation devices.

Our system overcomes several limitations of earlier magnetic neuromodulation platforms. Unlike previous approaches that mounted small electromagnets on the heads of experimental animals^26^, our system eliminates the need for tethering electromagnets, allowing fully untethered neuromodulation. Prior untethered designs, such as the CMA system, relied on rotating large 1 T NdFeB magnets (> 2000 cm³) using synchronized stepper motors^12^. While such systems avoided direct attachments, they required mechanically complex and expensive mechanisms and posed safety concerns. Large NdFeB magnets generate strong attractive forces that, if mishandled, pose risks of injury, equipment damage, and interference with electronics. Additionally, CMA-based systems typically generate only 20 mT at < 1 Hz, which is insufficient for many magnetic neuromodulation techniques (Table 1)^12^. In contrast, our system presented here offers better flexibility and supports higher MF densities (up to 60 mT) and frequencies (up to 20 Hz), in a modular, open-source format that reduces cost and complexity while remaining adaptable across various neuromodulation paradigms, including magnetomechanical and magnetogenetic applications. Although the solenoids demonstrated in this study generate up to 60 mT at 20 Hz, the modular design allows for scaling to higher MF densities and extended frequency ranges as needed for specific applications. This adaptability ensures the system can be optimized for a wide range of magnetic neuromodulation experiments, making it a versatile tool for neuroscience research.

Another integrated magnetic stimulation platform incorporating built-in magnetic and thermal sensors has been developed for low-field thoracic magnetic stimulation (LF-ThMS). LF-ThMS devices can deliver ~ 13 mT MF at 100*–*118 Hz, providing a cost-effective solution for pulsed magnetic stimulation applications^29^. While LF-ThMS is well-suited for its intended applications, its design utilizes a single power relay and a simplified circuit, which limits its flexibility in generating diverse magnetic stimulation patterns. In contrast, our system offers a broader range of MF intensities (6*–*60 mT) and frequencies (DC*-*200 Hz), allowing for greater adaptability across multiple experimental settings. Incorporating an H-bridge driver enables diverse stimulation waveforms, including bidirectional AMF, pulsed MF, and DC MF stimulation, making it particularly suitable for applications requiring precise and customizable magnetic neuromodulation control. Furthermore, the graphical user interface (GUI) enhances usability by providing researchers with an intuitive platform for designing, configuring, and monitoring stimulation protocols, reducing the technical barrier for users without an engineering background. In addition to LF-ThMS, several recently developed cost-effective and scalable magnetic stimulation systems have demonstrated the ability to generate MF intensities exceeding 50 mT at frequencies ranging from 5 to 20 Hz^9,10^. While these systems provide valuable contributions to magnetic neuromodulation research, they are often custom-built and require substantial engineering background for system assembly, coding, and experimental operation. Additionally, these systems lack a standardized and user-friendly interface, posing challenges for researchers without engineering expertise. In contrast, the system presented in this study is optimized for magnetic neuromodulation, offering enhanced flexibility, broader applicability, and a more comprehensive hardware*-*software integration. Its modular and open-source architecture ensures that higher MF intensities and frequencies can be easily implemented through different coil designs, making it a versatile and scalable tool for neuroscience research.

The integration of real-time sensor feedback and a closed-loop stimulation framework represents a major advancement of this system. Unlike conventional magnetic stimulation platforms, which operate with predefined, open-loop stimulation parameters, our system enables dynamic adjustments based on environmental and behavioral feedback. By incorporating video-based tracking, the system ensures MF application occurs only when the subject enters predefined target areas, preventing unintended stimulation outside experimental conditions. This feature is particularly beneficial in behavioral experiments, where field gradient variations near chamber boundaries could otherwise introduce confounding factors. Behavioral validation experiments, including light*-*dark box tests and real-time place preference tests, demonstrate that the system does not elicit aversive responses. Mice did not exhibit significant behavioral avoidance during MF exposure, confirming that the system operates without introducing stress or discomfort. Furthermore, the ability to monitor temperature, vibration, and noise in real time minimizes external influences, reducing the likelihood of unintended experimental artifacts. These capabilities make this system particularly well-suited for studying the effects of magnetic neuromodulation on freely moving animals while maintaining a high degree of experimental control.

Although the current sensor configuration is effective for real-time monitoring, researchers can expand the system’s sensing capabilities without modifying the console PCB circuit design and with only minimal adjustments to the Arduino firmware. If multiple digital sensors need to be connected, an I2 C multiplexer module (e.g., TCA9548 A) can enable seamless expansion while maintaining system simplicity and flexibility. Similarly, if additional analog sensors are required, an analog multiplexer module (e.g., CD74HC4051 or 74HC4067) can be used to increase the number of available analog inputs, enabling efficient data acquisition without altering the existing hardware design. These expansion options offer a straightforward pathway for integrating additional magnetic, temperature, or environmental sensors, ensuring that the system remains adaptable for diverse research applications.

While real-time sensor feedback and behavioral tracking enhance stimulation precision, waveform flexibility is generally not a major concern for most magnetic neuromodulation applications, as existing technologies primarily rely on square-wave MF/AMF. The system developed in this study is specifically designed to support these standard waveforms while maintaining simplicity and cost-effectiveness. To achieve this, the system incorporates an H-bridge driver, which was selected to efficiently generate square-wave MF/AMF, simplify the hardware design, and reduce overall cost, ensuring accessibility for a broad range of researchers. In the demonstrated magnetic apparatus, MF calibration was achieved by adjusting the voltage and current limits on the power supply, enabling precise regulation of MF density during application. Additionally, a single MF sensor continuously monitors the real-time MF intensity, ensuring field stability and preventing unintended fluctuations during experiments.

While this system is well-suited for a wide range of neuromodulation studies, it is currently limited in its ability to generate more complex waveforms, such as sinusoidal AMF. Implementing such waveforms requires additional function generators and power amplifiers to drive the solenoids, which would significantly increase system complexity and cost. Although the system adequately supports low-frequency magnetic neuromodulation techniques, it is not designed for magnetothermal stimulation, which requires high-frequency AMF in the 100 kHz to 1 MHz range. This limitation arises from the H-bridge driver and solenoid design, which are optimized for low-frequency applications (< 200 Hz). Implementing magnetothermal neuromodulation requires a full-bridge inverter circuit and specialized coil designs with resonant tank circuits capable of handling circulating currents exceeding 1 kA, significantly increasing both complexity and cost^30^. Despite this constraint, the system remains highly adaptable for other magnetic neuromodulation applications. Since most existing techniques operate at frequencies below 150 Hz, this system effectively supports magnetomechanical and magnetogenetic stimulation. Additionally, its modular design facilitates easy customization, allowing for the incorporation of higher MF intensities or alternative coil configurations as needed for specific applications.

Overall, this study presents a scalable, open-source, and cost-effective magnetic neuromodulation system that enhances accessibility, precision, and adaptability in neuroscience research. The system successfully integrates real-time sensor feedback, closed-loop control, and a modular coil design, making it suitable for both in vitro and in vivo applications. While the system is currently optimized for square-wave MF/AMF at frequencies up to 200 Hz, future improvements may focus on integrating programmable power supplies or current amplifiers to enable real-time field intensity adjustments. Despite its current limitations, the system’s flexibility and ease of modification position it as a valuable tool for advancing magnetic neuromodulation technologies. By addressing key challenges in cost, accessibility, and experimental control, this platform holds potential to accelerate progress in both basic and translational research.

## Materials and methods

### Hardware design

The PCBs were designed using EasyEDA, a free, web-based PCB design platform, and fabricated by the Sensing Electronic Workshop. Each PCB is a two-layer board with a total thickness of 1.6 mm. The console board with an Arduino Nano was designed for operation using an open-source GUI. The Arduino Nano generated 5 V digital signals, which were routed through NOT gates (SN74HC04 N, Texas Instruments) and AND gates (SN74HC08 N, Texas Instruments) to ensure phase-separated signal output. These signals were then passed through voltage followers implemented using TLC2272 operational amplifiers (Texas Instruments) to control the AMF in the solenoids via H-bridge drivers. The Arduino Nano provided 12 digital output pins (D2 to D13), five analog input/output pins (A1 to A5), and two additional analog input pins (A6 and A7). The digital output pins were organized into six pairs, each used to control one solenoid, allowing simultaneous generation of AMF in up to six solenoids. Each pair of digital pins was connected to 2 AND logic gates, 2 NOT logic gates, and 2 op-amps. There were 4 AND logic gates in an AND logic chip (SN74HC08 N, Texas Instruments), 6 NOT logic in a NOT logic chip (SN74HC04 N, Texas Instruments), and 2 op-amps in an op-amp chip (TLC2272, Texas Instruments). On the console board, a total of 3 AND gate chips, 2 NOT gate chips, and 6 op-amp chips (TLC2272) were used.

Analog pins A1 through A7 were allocated for various input and output functions. Pins A1 and A2 were configured as outputs to external devices. Pin A3 received input from the Hall sensor, A4 and A5 from the accelerometer, A6 from the temperature sensor, and A7 from the decibel sensor. The Arduino’s 5 V power rail supplied the Hall, decibel, and temperature sensors, while the 3.3 V output powered the accelerometer. Additionally, a 12 V external power supply was used to generate ± 6 V rails for the TLC2272 chips, stabilized with 1 kΩ resistors and 150 µF capacitors.

### Software design

The system in this study was compiled with Python 3.8.8. The GUI was developed with PyQt 5.15.7 library. The Arduino Nano was compiled with Arduino IDE 2.3.3. The code contains instructions for controlling the sensors and responding to signals received from Python. The Pyserial 3.5 library is used to connect Python to the system and the Arduino Nano via serial ports. In the magnetic system, the main Python module sends commands to the Arduino Nano specifying when to activate or deactivate the MF, and receives real time sensor values from the Arduino. The OpenCV 4.5.5.64 library is employed in the video module to perform real time video analysis during mouse behavioral tests. This module captures video frames from a live stream and analyzes mouse position by calculating the number of black pixels in the region of interest (ROI) between consecutive frames (see Algorithm for the real time mouse tracking section). After stimulation, the results, including real time measurements of MF strength, temperature, vibration, sound level (decibel), and mouse position, were saved as a “.csv” file. Further details on the software design are provided in the Results section.

### Algorithm for mouse tracking

Here, two video algorithms were used to detect the location of the mouse during magnetic stimulation for closed-loop stimulation. In the light*-*dark box test, we analyzed the area of the mouse body in the captured image to determine which chamber the mouse occupied during the experiment. First, the camera was set to capture the light box. The ROI in the image was drawn using the “Play Video” button in the first block of the “Camera” tab. The ROI image was converted to grayscale and smoothed using the median blur function in OpenCV. Next, the ROI image was converted to a binary image using the simple thresholding function in OpenCV with a threshold value of 127. When using black mice, such as C57BL/6 mice, against a white background, whether the mouse was in the ROI was determined based on the total number of black pixels. If the number of black pixels was below the threshold, the mouse was considered to be outside the ROI, indicating it was in the dark chamber. The pixel threshold could be adjusted using the “Threshold” input text box in the second block of the “Camera” tab. During the experiment, the real time video feed shown in the second block of the “Camera” tab included the current number of black pixels in the ROI, the detected chamber location, and the duration of time spent in each chamber.

In the 3-chamber place preference test, mouse location was determined using an algorithm similar to that of the light-dark box test. Whether the mouse was inside the middle transparent chamber was analyzed based on the area of the mouse’s body in the image of the middle chamber. When the mouse exited the middle chamber, its entry into either side chamber was inferred from its last known position in the previous frame. First, the camera was set to capture the middle chamber. Two ROIs were drawn on each side of the middle chamber using the “Play Video” button in the first block of the “Camera” tab. The images in both ROIs were converted to grayscale and smoothed using the median blur function. Binary images were then created using the OpenCV thresholding function at a threshold of 127. When using C57BL/6 mice against a white background, the presence of the mouse in either ROI was determined by the total number of black pixels. The threshold values for the two ROIs could be configured separately using the “Threshold” input boxes in the second block of the “Camera” tab. If the number of black pixels in both ROIs was below their respective thresholds, the mouse was considered to be in one of the side chambers. To determine which side, the previous frame was referenced: if the mouse had been in the ROI closer to the right chamber, it was classified as entering the right chamber, and vice versa. During the experiment, the real time video panel in the second block of the “Camera” tab displayed current black pixel counts, detected chamber, and time spent in each chamber. Plots in the fourth block of the “Camera” tab also visualized stimulation status and chamber transitions. Timing and duration data were saved to a “.csv” file at the end of each experiment. This algorithm allowed accurate chamber detection even when coils obstructed direct visual access, supporting closed-loop magnetic stimulation in the current study. In the freely moving test, the mouse’s speed and total distance traveled were recorded and analyzed using Python 3.8.8 and OpenCV 4.5.5.64. The mouse’s body center was detected in each frame. The total distance and speed were calculated based on the displacement of this center across consecutive frames.

### FEMM simulation

The software Finite Element Method Magnetics (FEMM, version 4.2) was used to perform two-dimensional axisymmetric magnetic simulations to calculate the MF generated by the different solenoids in this study. The properties of copper wire (12 or 18 AWG) and air in the materials library of FEMM 4.2 were used. In the axisymmetric simulation for the coils used with the 24-well dish, two square cross-sections were modeled. Each cross-section measured 10 cm in length, 1.5 cm in width, and was located 5.5 cm from the central axis. Each was filled with 1500 turns of 18 AWG copper wire.

In the simulation of the 3.5 cm coil for fluorescence microscopy, the coil cross-section was modeled as a square shape with a height of 4 cm, a width of 5 cm, and located 1.75 cm from the central axis. The simulation used 2000 turns of 18 AWG copper wire. For the 10 cm coil used in the mouse behavioral test, the cross-section was defined as a square with a height of 4 cm, a width of 5 cm, and positioned 5.5 cm from the central axis. This model used 500 turns of 12 AWG copper wire. To generate a homogeneous MF, four 10 cm coils were aligned horizontally along the central axis, with 1 cm gaps between coils in a group and 2 cm gaps between groups.

For the 20 cm coil used in the mouse open-field test, the coil cross-section was modeled as a square with a height of 5 cm, a width of 4 cm, and positioned 10.5 cm from the central axis. This configuration also used 500 turns of 12 AWG copper wire. To achieve a homogeneous MF, six 20 cm coils were aligned horizontally along the central axis, using 1 cm spacing between coils within each group and 2 cm spacing between groups.

The homogeneity of MF in FEMM simulation was calculated by the following equation:

1 $$\:Homogeneity=\:\frac{{B}_{max}-{B}_{min}}{{B}_{mean}}\times\:100\%$$

Where B_max_ is the maximum MF in the given range, B_min_ is the minimum MF in the given range, and Bmean is the average MF within the given range.

### Solenoid fabrication

The coils for the 24-well dish consisted of two identical coils. Both were built on rectangular cubic acrylic molds measuring 10 cm in length, 9.1 cm in width, 3.6 cm in height, and 0.3 cm in thickness. The coils were built with 18 AWG copper wire. The acrylic mold was retained after coil construction. To fit the 24-well culture dish, which measures 12.7 cm in length, at the center of the two coils, each coil included two vertical acrylic stripes positioned 6.7 cm from the edge of the mold. The coils with 3.5 cm inner diameter were built on a cylindrical metal mold measuring 4 cm in height and 3.5 cm in diameter. These coils were also constructed using 18 AWG copper wire, and the mold was removed after winding. The completed coils were then secured with zip ties. The coils for the 10 cm arena were constructed with 12 AWG copper wire on an acrylic mold featuring 0.5 cm thickness at both ends and 0.2 cm thickness at the center. This acrylic mold contained a hollow cylinder with an inner diameter of 11 cm and height of 4 cm. The mold remained in place after coil construction, and zip ties were used to secure the coil. Similarly, the coils for the 20 cm arena were built with 12 AWG copper wire on an acrylic mold with 0.5 cm thickness at the edges and 0.2 cm at the center. This mold featured a hollow cylinder with a 21 cm inner diameter and a height of 5 cm. The mold was left in place after winding, and the coils were secured with zip ties.

### Measurement of MF

A Gauss meter (TM801, KANETEC) was used to measure the distribution of MF density within the coil. The probe was 1 cm in diameter and was aligned with the MF during measurement. The coils for the 24-well dish were designed to fit a 24-well plate measuring 2 cm in height, 12.7 cm in length, and 8.5 cm in width, positioned at the center of the coil. When placing the culture dish inside the coils, the distance from the inner wall of the long side of the coil to the long side of the dish was 0.5 cm, and the distance from the short edge of the coil to the short side of the dish was 3.65 cm. The center of the corner well was located 1.9 cm from the short edge and 1.6 cm from the long edge of the culture dish. To measure the MF strength at each well of the culture dish, we first measured the MF at 5.55 cm from the short edge, 2.3 cm from the long edge, and 1 cm in height from the bottom of the coils. The distance between each well was 1.8 cm in both directions. MF intensity at all 24 wells arranged in 6 columns and 4 rows was measured. For the coil with a 3.5 cm inner diameter, the coil with a 10 cm arena, and the coil with a 20 cm arena, the probe was placed at 1 cm intervals from the center of the coils to the inner wall. If the probe reached a position within 1 cm of the coil wall, it was placed directly against the wall. Because the MF was asymmetrical, measurements were only performed in the sagittal plane. Measurements for the coil with a 3.5 cm inner diameter included 3 columns and 4 rows at the center sagittal plane, covering 4 cm in width and 3 cm in height. The coil for the 10 cm arena was measured at 9 columns and 21 rows, covering 10 cm in width and 21 cm in height. The coil for the 20 cm arena was measured at 19 columns and 35 rows, covering 20 cm in width and 35 cm in height. MF homogeneity was calculated using the same Eq. (1) as used for MF homogeneity in the FEMM simulation.

### Sensors calibration

Four sensors were used in this study, including the Hall sensor, temperature sensor, acceleration sensor, and decibel sensor. The precision of these sensors was calibrated using commercial handheld devices. The Hall sensor (SS495 A, Honeywell) was tested using an electromagnet (10.5 W, Longsource) measuring 3 cm in height and 6.5 cm in diameter. The electromagnet generated an AMF with 30 s “off” and 30 s “on” periods for 5 cycles. The Hall sensor and the probe of the handheld Gauss meter (FM302, Projekt Elektronik) were placed side-by-side near the electromagnet to measure MF strength. A 100 ml glass beaker filled with 40 °C water was used to test the temperature sensor (TMP36GT9Z, Analog Devices). The sensor was attached to the outer wall of the beaker, while an infrared thermometer (IR-829, Phils Trust Medical Center) was positioned on the side. Ice cubes were added during the test to decrease the temperature gradually. The acceleration sensor (ADXL345, Analog Devices) was tested using a motor. The sensor and a handheld vibrometer (GM63 A, Benetech) were placed on the motor, which was manually turned on and off four times during the test. A cellphone speaker was used to test the decibel sensor (Gravity, TaiwanIOT). The sensor and a handheld decibel meter (UT353, Uni-T) were positioned at equal distances from the speaker. A 10 Hz square wave audio signal was played during the test, and the volume was manually increased.

### Standard usability test

Researchers and students with varying technical backgrounds were recruited to evaluate the system’s usability. After a brief tutorial, participants completed key tasks such as setting up and calibrating the system, configuring stimulation parameters via the GUI, monitoring real time sensor feedback, and performing data acquisition. Usability was assessed using the System Usability Scale (SUS), a 10-question Likert-scale survey (1*–*5) measuring ease of use, clarity, efficiency, and satisfaction. Responses were converted to individual scores following the SUS scoring method, where odd-numbered question scores were adjusted by subtracting 1, and even-numbered question scores were adjusted by subtracting from 5. The sum was then multiplied by 2.5 to obtain a final score ranging from 0 to 100. Scores above 68 indicate above-average usability, while scores exceeding 80 suggest a highly usable system. Qualitative feedback was analyzed to identify usability concerns and potential improvements.

### Animal experiments

This study is performed in accordance with relevant guidelines and regulations. All methods are reported in accordance with ARRIVE guidelines (Animal Research: Reporting of In vivo Experiments). All animal procedures were approved by the Institutional Animal Care and Use Committee (IACUC) at National Yang-Ming Chiao Tung University (NYCU), following the NYCU Guide for the Care and Use of Laboratory Animals. Mice were purchased from LASCO and housed under a 12-hour light*-*dark cycle at the NYCU Laboratory Animal Center before experimentation. Only 8- to 12-week-old male C57BL/6 mice were used for in vivo studies.

We performed three behavioral tests on the mice: the light*-*dark box test, the place preference test, and the freely moving test. The light*-*dark box consisted of two chambers, one illuminated and one dark. Both chambers were horizontal cylindrical boxes 14 cm in length and 10 cm in diameter, with a floor plate measuring 8 cm in width and 14 cm in length. The two chambers were connected by a square door, 3 cm high and 4 cm wide. A 100 W lamp was positioned above the light chamber, while the dark chamber was covered with black tape and placed at the center of the 10 cm solenoids. On the first day, the mouse was allowed to habituate in the light*-*dark box for 1 h without stimulation. After habituation, the mouse was observed for a 5-minute pre-stimulation period without magnetic stimulation but with sensors and video recording. On the second day, the mouse was placed back into the same chamber for magnetic stimulation. The stimulation protocol was 30 s MF off and 30 s MF on for five cycles. The AMF strength and frequency were 50 mT and 10 Hz. At the start of the test, the mouse was placed in the light chamber, and the stimulation protocol was initiated when the mouse entered the dark chamber for the first time. Based on the real time video feedback, AMF was only activated when the mouse was in the dark chamber during the 30-second “MF on” phase.

The place preference test utilized a box with three chambers, all of which were horizontal cylindrical boxes. The middle transparent chamber was 20 cm in length and 10 cm in diameter. The side chambers were 14 cm long and 10 cm in diameter, covered with black tape. The floor plates of all chambers were 8 cm wide with different floor patterns. On the first day, the mouse was habituated to the test box for 1 h without stimulation. After habituation, the mouse was observed for a 10-minute pre-stimulation period without magnetic stimulation but with sensors and video recording. On the second day, the mouse was placed in the middle chamber without access to the side chambers for 10 min. Next, the mouse was allowed to explore all three chambers with magnetic stimulation. The stimulation protocol was 30 s AMF off and 30 s AMF on for ten cycles. The AMF strength and frequency were 50 mT and 10 Hz. Magnetic stimulation began once the mouse had traversed all three chambers and returned to the middle chamber. Based on the real time video feedback, AMF was activated only when the mouse was in the stimulation chamber during the 30-second “MF on” phase.

For the freely moving test, the mouse was placed in a vertical cylindrical box measuring 14 cm in height and 10 cm in diameter, covered with white tape. The box contained 1*–*2 cm of fresh bedding. Before stimulation, the mouse was habituated in the box for 1 h. Then, magnetic stimulation was applied for 10 min. The stimulation protocol was 30 s AMF off and 30 s AMF on for ten cycles.

### Statistical analysis

All statistical analyses were performed in JASP (v0.16.2.0, JASP team) and Python (3.8.8). All error bars in the dot plots indicate the standard error of the mean (s.e.m.). The numbers of individual cells, samples (individual cultures), and animals in each experiment are indicated in the figure and table legends. The Wilcoxon signed-rank test was used for paired comparisons. Pearson’s correlation and Bland*-*Altman analysis were used to evaluate agreement and bias between measurements.

## Electronic supplementary material

Below is the link to the electronic supplementary material.

Supplementary Material 1

## Data Availability

The data are available upon request from the corresponding author. The code in this study is accessible in the code repository (https://github.com/phclab/magnetic_system.git).
