# Supplementary material for: Open-source magnetic system for wireless neuromodulations in vitro and for untethered brain stimulation in vivo
Source: Sci Rep. 2025 May 22;15:17814. doi: 10.1038/s41598-025-03076-7 (PMC12098840; doi:10.1038/s41598-025-03076-7)
Supplement: Supplementary file 1 — Supplementary Material 1 [file 41598_2025_3076_MOESM1_ESM.docx]

**Supplementary Information**

**Open-source magnetic system for wireless neuromodulations *in vitro* and for untethered brain stimulation *in vivo***

Jun-Xuan Huang^1^, Ping-Hsiang Yen^1^, Chao-Chun Cheng^1^, Yi-Cheng Fang^1^, Po-Han Chiang^1^*

^1^Institute of Biomedical Engineering, National Yang Ming Chiao Tung University, Taiwan (R.O.C.)

*corresponding author Email: phc@nycu.edu.tw

**List of content:**

**Supplementary Methods**

**Supplementary Table S1 to S5**

**Supplementary Figure S1 to S2**

**Supplementary Methods**

**Magnetic field calculation of circular multilayer coils**

The magnetic field along the central axis of a current-carrying circular coil can be derived from the Biot-Savart Law, which describes the contribution of an infinitesimal current element to the total magnetic field. For a single circular current loop with a radius R, carrying a current I, the axial magnetic field at a distance z from the center of the loop is given by:

$$B=\frac{\mu_{0}IR^{2}}{2{(R^{2}+z^{2})}^{3/2}}$$

where μ_0_​ is the permeability of free space (4π×10^−7^ T·m/A). In the case of a coil with multiple turns, the total field is obtained by summing the contributions from all turns.

A practical coil consists of multiple layers of turns stacked radially outward from the inner radius R_in_​. The number of turns per unit width (turn density) is given by

$$n_{turn}=\frac{N}{w}$$

where N is the total number of turns, and w is the coil width. The effective radius for each layer is given by

$$R_{i}=R_{in}+i\cdot d$$

where d is the wire diameter and i is the layer index. Since the coil has a finite width, its field contribution at a position z along the axis is computed by integrating over all turns.

For a coil of finite width, the total axial magnetic field at a given position is calculated by summing the contributions from all layers. The field from a single layer at an axial distance z is given by:

$$B_{i}=\frac{\mu_{0}In_{turn}}{2}\left( \frac{z-z_{1}}{\sqrt{\left( z-z_{1} \right)^{2}+{R_{i}}^{2}}}-\frac{z-z_{2}}{\sqrt{\left( z-z_{2} \right)^{2}+{R_{i}}^{2}}} \right)$$

where z_1_ = −w/2 and z_2_ = w/2 represent the axial boundaries of the coil. The total magnetic field at the given position is then obtained by summing the contributions from all layers:

$$B_{coil}=\sum_{i=1}^{N_{layers}} B_{i}$$

where N_layers_​ represents the total number of radial layers. This formulation accounts for the full three-dimensional structure of the coil and ensures an accurate calculation of the field at any given axial position.

When multiple coils are arranged along the same axis, the total magnetic field at a given position is obtained by summing the contributions from each individual coil. Each coil generates a field that follows the Biot-Savart law, and since the coils are spaced apart by specified gaps, their fields are superimposed at the desired calculation point.

For a system with coils number of N_coils_ , each coil is positioned at a different center location z_j_​, where j denotes the coil index. The center positions of the coils are determined by the sum of the coil heights and the inter-coil gaps, starting from the first coil’s reference position. The axial magnetic field contribution from each coil at a given position zzz is calculated using the same finite-width coil formulation:

$$B_{i}=\frac{\mu_{0}In_{turn}}{2}\left( \frac{z-\left( z_{j}-w/2 \right)}{\sqrt{\left( z-\left( z_{j}-w/2 \right) \right)^{2}+{R_{i}}^{2}}}-\frac{z-\left( z_{j}+w/2 \right)}{\sqrt{\left( z-\left( z_{j}+w/2 \right) \right)^{2}+{R_{i}}^{2}}} \right)$$

$$B_{i}=\sum_{j=1}^{N_{layers}} B_{j}$$

where z_j_​ is the center position of the j-th coil, w is the coil width, and R_i_ is the effective radius of each layer within the coil.

The total magnetic field at the desired position z along the axis is obtained by summing the fields from all coils:

$$B_{total}=\sum_{i=1}^{N_{coils}} B_{i}$$

This formulation ensures that the contributions from all coils are correctly accounted for, incorporating their individual positions along the axis. Since the field contributions decrease with distance from the coil, coils farther from the calculation point contribute less to the total field. This method allows accurate prediction of the magnetic field for systems with multiple coils.

**Magnetic field calculation of rectangular multilayer coil**

Similar to circular coils, the magnetic field generated by a rectangular coil along its central axis can be derived using the Biot-Savart Law, considering the contributions from each segment of the coil. Unlike circular coils with a symmetrical field distribution, rectangular coils consist of distinct field contributions from their longer and shorter sides. This asymmetry requires separate calculations for each segment to determine the total field at a given point.

For a rectangular coil with a long side of length **L**, a short side of length **W**, and a total of **N** turns carrying a current **I**, the total magnetic field at the center can be determined by summing the contributions from all segments. The field generated by a single wire segment along one side is obtained from the Biot-Savart Law, which states that the magnetic field at a perpendicular distance rrr from the center of a finite straight wire of length **l** is given by:

$$B=\frac{\mu_{0}I}{2\pi}\cdot\frac{l/2}{r\sqrt{{(l/2)}^{2}+r^{2}}}$$

Applying this equation to the rectangular coil, the total field at the center is given by the sum of the contributions from the two long sides and the two short sides. Specifically, the field at the center of a rectangular coil with length L and width W is:

$$B=2\left( \frac{\mu_{0}I}{2\pi}\cdot\frac{L/2}{r_{L}\sqrt{{(L/2)}^{2}+{r_{L}}^{2}}} \right)+2\left( \frac{\mu_{0}I}{2\pi}\cdot\frac{W/2}{r_{w}\sqrt{{(W/2)}^{2}+{r_{w}}^{2}}} \right)$$

where **r_L_**​ and **r_W_**​ represent the perpendicular distances from the center of the coil to the long and short sides, respectively.

When the coil consists of multiple turns and the wire has a finite thickness d, the position of each turn must be considered to account for its offset from the center. When magnetic field is evaluated at a distance **z** from the end of a multilayer coil, the perpendicular distance **r_L_**​ and **r_W_**​ of the calculation point to the **j-th** turn in the **i-th** layer is:

$$r_{L}=\sqrt{{(jd+z)}^{2}+\left( \frac{W}{2}+id \right)^{2}}$$

$$r_{w}=\sqrt{{(jd+z)}^{2}+\left( \frac{L}{2}+id \right)^{2}}$$

where **d** represents the diameter of the wire. This correction accounts for the displacement of each turn from the center due to the winding structure.

To compute the total field in a multi-layer coil, the field contributions from all turns in all layers must be summed. The total magnetic field at the center of a multi-layer coil is expressed as:

$$B_{total}=\sum_{i=1}^{N_{layers}} \sum_{j=1}^{N_{turns}} B_{j}$$

where N_layers_​ represents the number of layers in the coil, and N_turns_ represents the number of turns in each layer. This summation approach ensures that the effects of the finite wire thickness and multi-layer structure are incorporated into the final field calculation.

This formulation provides a more accurate representation of the magnetic field at the center of a rectangular coil by considering the geometric distribution of the turns. It extends the basic Biot-Savart model to account for practical constraints such as wire thickness and multi-layer windings, which are critical in designing electromagnets and inductive systems.

**Supplementary Tables**

| System | item | Price (USD) | Quantity |
| --- | --- | --- | --- |
| CMA system for Magnetomechanical Stimulation ^12^ | L611 Precision Rotation Stage (Physik Instrumente) | ~$9900 | 1 |
|  | NdFeB (12.5 cm × 15 cm × 12.5 cm) | ~$3600 | 10 |
|  | 300 mm vertical translation stage | ~$5000 | 1 |
| System for Magnetothermal Stimulation ^2-4^ | Keysight, 33210A Function Generator | ~$1500 | 1 |
|  | Keysight DC Supply (600V, 2.6A) | ~$5100 | 4 |

**Table S1. The main cost of other magnetic neural experiments in USD.** Only devices over $1000 were listed. Custom devices, such as high-power inverter, cooling system, and other components were not listed here.

| Model | Manufacturer | MaximumVoltage | Maximum Current | Power | Price  (USD) |
| --- | --- | --- | --- | --- | --- |
| HJS-1000-0-48V/20A (power supply) | Yueqing Zhijiu Electric Co., Ltd. | 48V | 20A | 960W | $88.43 |
| AQMH3615NS 2.0 (H-bridge) | Akelc | 36V | 12A (without heat dissipation),  15A (with simple heat dissipation),  20A (with thick large heat sink); instant peak current 110A | 380W | $10.76 |

**Table S2. Power Supply and H-Bridge.** The manufacturer, voltage, current, power and price of power supply and H-bridge.

| Name | Quantity | Manufacturer | Price  (USD) |
| --- | --- | --- | --- |
| Female Header Connector | 2 | ZHOURI | 0.133 |
| DC Power Socket | 1 | XKB Connectivity | 1.132 |
| 150uF Capacitor | 2 |  | 1.67 |
| 1k Resistor | 2 |  | 0.11 |
| SN74HC04N | 2 | Texas Instruments | 0.559 |
| SN74HC08N | 3 | Texas Instruments | 0.551 |
| TLC2272CP | 6 | Texas Instruments | 6.176 |
| JST Male Connector | 6 |  | 0.21 |
| Arduino Nano | 1 | Arduino | 19.99 |
| Power Supply | 6 | Yueqing Zhijiu Electric Co., Ltd. | 88.43 |
| H-Bridge | 6 | Akelc | 10.76 |
| SS495A | 1 | Honeywell | 4.24 |
| TMP36GT9Z | 1 | Analog Devices | 1.56 |
| SEN0232 | 1 | TaiwanIOT | 37.76 |
| ADXL345 | 1 | Analog Devices | 1.83 |

**Table S3. Components in the console board List.** The quantity, manufacturer, and price of each component on the console board, sensor, H-bridge, and power supply.

|  | Range |
| --- | --- |
| Protocol Steps | 30 Steps |
| Duration per Step | 0-3600 sec |
| Frequency per Step | 0-500 Hz  (0-200Hz with sensors, 0-500Hz without sensors) |
| Deadtime per Step | 0-100 ms |

**Table S4. The range of each parameter in the system.** The setting range of step, duration, frequency, deadtime in the protocol and the output range of the console board signal and the H-bridge driver.

| **Index** | **Question** | **Strongly Strongly Disagree Agree** | | | | |
| --- | --- | --- | --- | --- | --- | --- |
|  |  | **1** | **2** | **3** | **4** | **5** |
| **1** | I think that I would like to use this system frequently. |  |  |  |  |  |
| **2** | I found the system unnecessarily complex. |  |  |  |  |  |
| **3** | I thought the system was easy to use. |  |  |  |  |  |
| **4** | I think that I would need the support of a technical person to be able to use this system. |  |  |  |  |  |
| **5** | I found the various functions in this system were well integrated. |  |  |  |  |  |
| **6** | I thought there was too much inconsistency in this system. |  |  |  |  |  |
| **7** | I would imagine that most people would learn to use this system very quickly. |  |  |  |  |  |
| **8** | I found the system very cumbersome to use. |  |  |  |  |  |
| **9** | I felt very confident using the system. |  |  |  |  |  |
| **10** | I needed to learn a lot of things before I could get going with this system. |  |  |  |  |  |

**Table S5.** **Standard usability survey.**

|  | 24well coil | 3.5cm coil | 10cm coil | 20cm coil |
| --- | --- | --- | --- | --- |
| Real Coil Inner Radius (mm) | 36 x 92 | 35 | 110 | 210 |
| Coil Height (mm) | 100 | 40 | 40 | 40 |
| Copper Wire Diameter with Insulation(mm) | 1.09474  (18 AWG) | 1.09474  (18 AWG) | 2.13868  (12 AWG) | 2.13868  (12 AWG) |
| Copper Wire Diameter without Insulation:(mm) | 1.02362  (18 AWG) | 1.02362  (18 AWG) | 2.05232  (12 AWG) | 2.05232  (12 AWG) |
| Number of Turns | 1500 | 2000 | 420 | 400 |
| Coil Current (A) | 3.2 | 2.7 | 7.2 | 9.5 |
| Frequency (Hz) | 10 | 10 | 10 | 10 |
| Number of coils | 2 | 1 | 4 | 6 |
| Gaps between coils (mm) | 10 | - | 10, 20, 10 | 10, 20, 10, 20, 10 |
| Magnetic Field (mT) | 64.75 | 55.28 | 56.17 | 68.20 |

**Table S6. Parameters and result of magnetic field calculation by Biot-Savart Law.** The inner radius, length, wire diameter, number of turns, current, frequency, height, resistance, inductance, and total impedance of each coil. The number of turns were estimated by the resistance of the coil.

**Supplementary Figures**


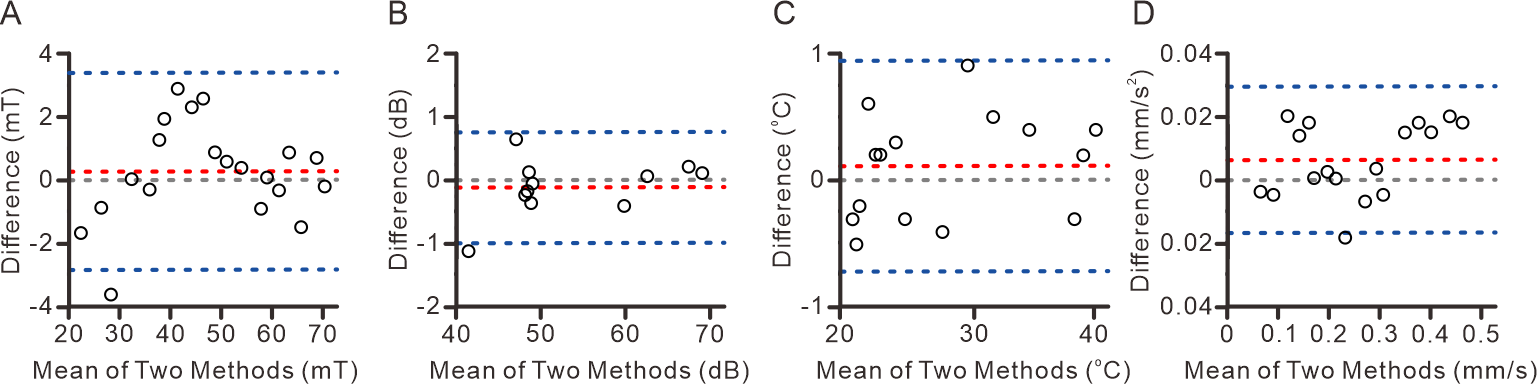


**Figure S1.** **The Bland-Altman analysis between each sensor and the corresponding meter.** (A) The Bland-Altman analysis between Hall sensor and the handheld Gauss meter. (Upper LoA = 3.33, Lower LoA = -2.86, Bias = 0.24) (B) The Bland-Altman analysis between the decibel sensor and the handheld decibel meter. (Upper LoA = 0.77, Lower LoA = -0.98, Bias = -0.11) (C) The Bland-Altman analysis between the temperature sensor and the infrared thermometer. (Upper LoA = 0.94, Lower LoA = -0.71, Bias = 0.11) (D) The Bland-Altman analysis between the acceleration sensor and the handheld vibrometer. (Upper LoA = 0.03, Lower LoA = -0.02, Bias = 0.01)


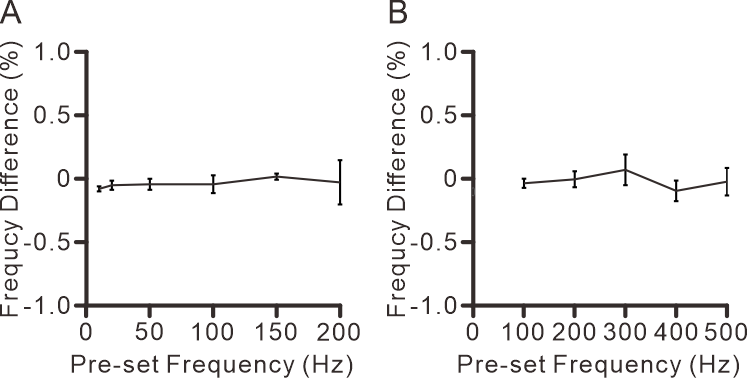


**Figure S2. The difference between pre-set and real-time frequency in each frequency.** (A) The frequency difference when 4 sensors’ detections were switched on. (B) The frequency difference when all sensors detections were switched off.
